# Segmentation of mine overburden dump particles from images using Mask R CNN

**DOI:** 10.1038/s41598-023-28586-0

**Published:** 2023-02-04

**Authors:** Shubham Shrivastava, Sudipta Bhattacharjee, Debasis Deb

**Affiliations:** Department of Mining Engineering, Indian Institute of Technology Kharagpur, Kharagpur, West Medinipur, 721302 West Bengal India

**Keywords:** Machine learning, Civil engineering

## Abstract

The stability of mine overburden dumps is crucial for the efficient operation of mining industries. The size distribution of particles affects the shear strength of dump slopes. Identification of dump particles from images is challenging as they vary in size, shape, color, granularity, and texture. In this paper, a unique way of identifying the particles from dump images using Artificial Intelligence is presented that can be used to determine the particle size distribution of dump. Mask R CNN with ResNet50 plus an FPN as a backbone network which is the current state of the art for instance segmentation has been implemented to segment the particles from dump images at detailed pixel level and to obtain their boundary. Experimental results showed promising results to delineate the particles and obtain masks over them. Our model has achieved a training accuracy of 97.2% for the dataset containing 31,505 particles. The model predicted the areas of dump particles with a mean percentage error of 0.39% and a standard deviation of 0.25 when compared to the ground truth values. The calculation of coordinates of the detected boundaries using the model significantly reduces the time and effort that are generally put in rock mechanics laboratories.

## Introduction

During surface mining operations of coal and other ore deposits, a huge amount of earth material (soil-rock mixture) is removed and stacked on a limited land area acquired by the management. This earth material constitutes particles ranging from clay to big boulders, and it is referred to as mine overburden dumps^1–3^. With the increase in production capacity of open pit mines, millions of cubic metres of overburden are removed every year^4^. Hence, maintaining the stability of such massive structures plays a vital role in the proper functioning of mining activities. Shear strength of dumps can be defined by cohesion (c) and angle of internal function ($\phi$)^3,5^. These two parameters c and $\phi$ are controlled mainly by the size distribution of dump particles^6,7^. The traditional method of determining the particle size distribution of the sample of dump material is through sieve analysis^7,8^. However, in recent years, many works have been carried out to estimate the particle size distribution from dump images.^9–11^.

Identification of dump particles from the images is a challenging task as the dump images are affected by various natural conditions like the intensity of daylight, presence of moisture in the dump particles, occlusion of dump particles^12^. Many industrialists have used blast fragmentation software such as Split, Fragscan, Wipfrag^13,14^ etc. to obtain particle size distribution curves. Researchers have tried to detect the particles using edge detection techniques such as Sobel, Canny filters. Morphological operations have also made it possible to segment the particles^12^. But in most of the cases, images contain few particles that are distinct from each other, and these methods are based on color contrast. The in situ particle identification is still the area where a small light has been thrown. On the field, the particles vary a lot in terms of their texture, shape, color, granularity, which makes them even more difficult to detect. Segmentation of such multi-scale and multi-shape objects using traditional image processing techniques seems to be challenging.

Convolutional Neural Networks (CNN) are nowadays used heavily in the field of computer vision such as autonomous driving, medical imaging etc^15,16^. High accuracy has been found in the area of image classification, object detection, and segmentation^17–19^. It is now possible to locate the objects of different classes in the images and predict the coordinates of bounding boxes. The structure of CNN consists of other convolution and max pooling layers along with some methods of regularization. The features are learnt during the forward and backward steps of training. Initial layers are responsible for learning lower level features such as sides, edges and these lower level features are used to learn higher-level features that describe target classes from the deep layers^20^. The learning of the model takes place by updating the weight and bias coefficients. Karimpouli and Tahmasebi (2019)^21^ applied CNN autoencoder network that was used to semantically segment the rock particles that were similar in scale and size for the study of digital rock physics. For the granularity analysis of rock images, Cheng and Guo (2017)^22^ implemented CNN with 4 convolutional and 2 fully connected layers.

In this paper, Mask R CNN^23^ which is the current state of the art in instance segmentation has been implemented. The developed model predicts masks along with the bounding boxes. The location and size of particles in the dump images can be tracked down, which can be used to generate particle size distribution curves (this will be the next step of study). This paper is organised in the following manner: (1) Materials and methods which describe image acquisition, dataset preparation, and architecture of Mask R CNN. (2) Results that describe the experimental details and result, and (3) Discussion and future scope of the work.

## Materials and methods

### Image acquisition

**Figure 1:**
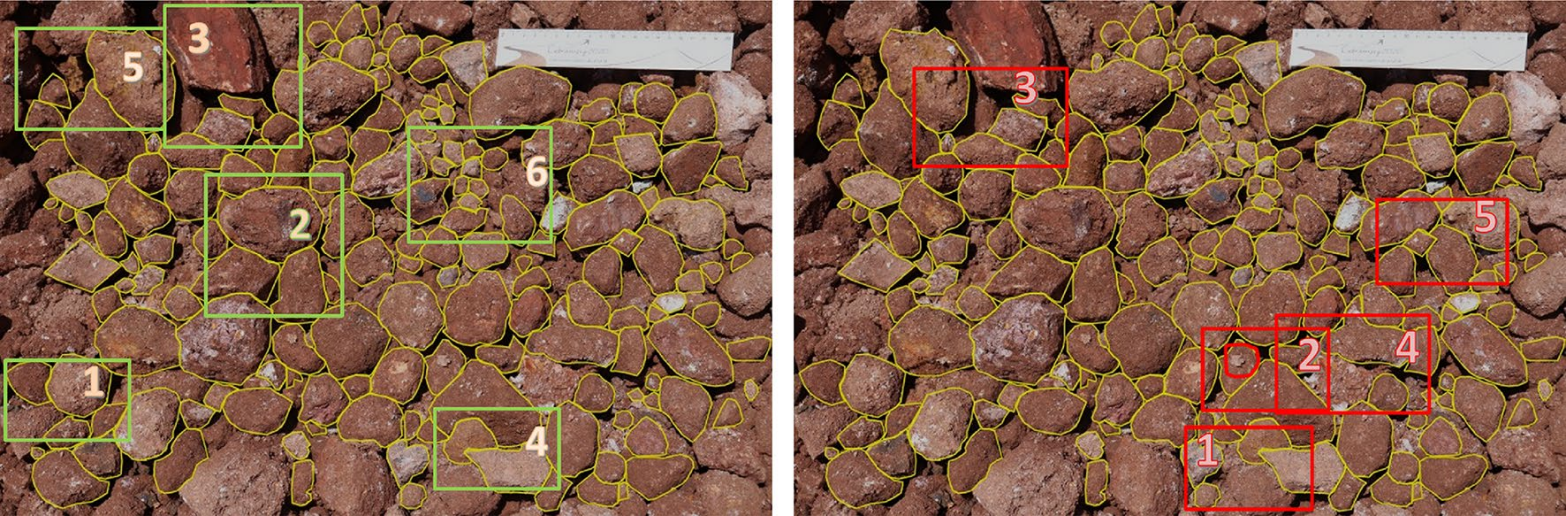
Qualitative guidelines for annotation and labelling of dump particles from images

For the purpose of developing a Mask R CNN based model, 500 images of different sections of an active overburden dump of JSPL Iron ore mine, Tensa, Sundargarh, Orissa, India, had been collected on the month of January 2020. The dump particles mainly consisted of red, yellow, and brown shale. Size of particles varied from clay to boulder size. Quantity of fine particles in some images are more than 70% and in most of the images are evenly distributed. In this context, we describe the fine particles as the regions of dump images which are smooth and where the particle boundaries are not clearly distinct. Images were captured to obtain mixed proportions of different colored shales. To convert the image pixels into real world dimension coordinates, a white-colored labelled scale was put on the a face of dump at the corners when taking images (Fig. 1). This small trick can later be helpful at the time of determining particle size distribution curve. Images were taken from various distances perpendicular to the face of dump so that the number of particles in an image varies from 200 to more than 400 in some cases. Images were captured properly to make sure it were distortionless. In the presence of moisture content, particles occluded, and their boundaries were not distinct in some cases. Images were taken at different times in a day. As a result, the shadows of dump particles affected a few neighboring particles. 31,505 particles were extracted from the images. The resolution of all images is 6000 $\times$ 4000 and were captured by Canon EOS 200D digital SLR camera. Dump image dataset was fed to the model without applying any morphological operations so that the model can identify dump particles from in situ condition.

### Dataset annotation and labelling

Dump images contain particles that vary widely in size. Annotating each particle in an image seems to be challenging. Therefore, the task of annotation and labelling has been confined to some ground rules which the authors prepared after considering the whole image dataset. Figure 1 shows dumps images that mention do’s and don’ts in green and red boxes respectively.

Do’s:

1. Only those particles are annotated which are lying in the image boundary whose surface edges are clearly defined.
2. Mask boundaries of two particles should be in contact, if they are touching each other.
3. Mask should be drawn to cover the depth of particle entirely, if clearly visible.
4. If two particles lie above one another, then bottom particle should also be annotated keeping in mind the two dimensional view of the dump particles.
5. The rock particles which are slightly blurred are also annotated.
6. Fine particles are annotated only if their boundary can be defined.

Don’ts:

1. Annotate the particles at the boundary of image whose dimension cannot be estimated.
2. Annotate the fine particles (dust and sand) lying over coarse rock particles.
3. Annotate the particles whose dimensions can’t be speculated due to shadows.
4. Annotate the particles which are lying below many particles and their dimensions can’t be defined.
5. Intersect the mask boundaries.

**Figure 2:**
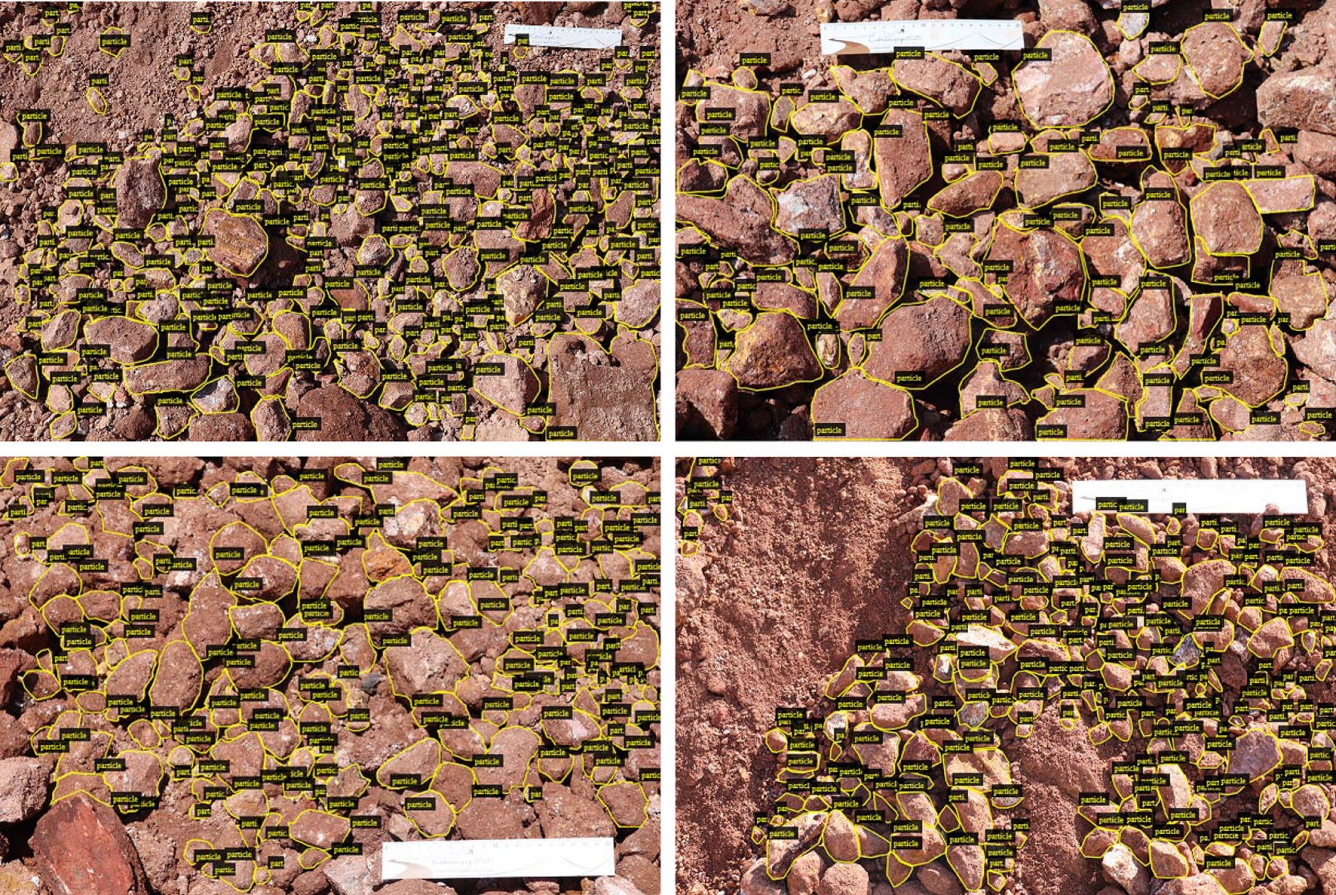
Some examples of annotated and labelled images for training dataset

VGG Image Annotator (VIA)^24^ version 2.0.8 which is developed by Visual Geometry Group, Department of Engineering Science, University of Oxford has been used to annotate and label the dump particles. It is a manual standalone software that runs in a web browser and it allows to export the annotations in COCO^25^ format. In Fig. 2, some examples of annotated and labelled dataset are shown. A single class named “particle” was selected as an attribute for the purpose of labelling.

### Model structure of Mask R CNN

**Figure 3:**
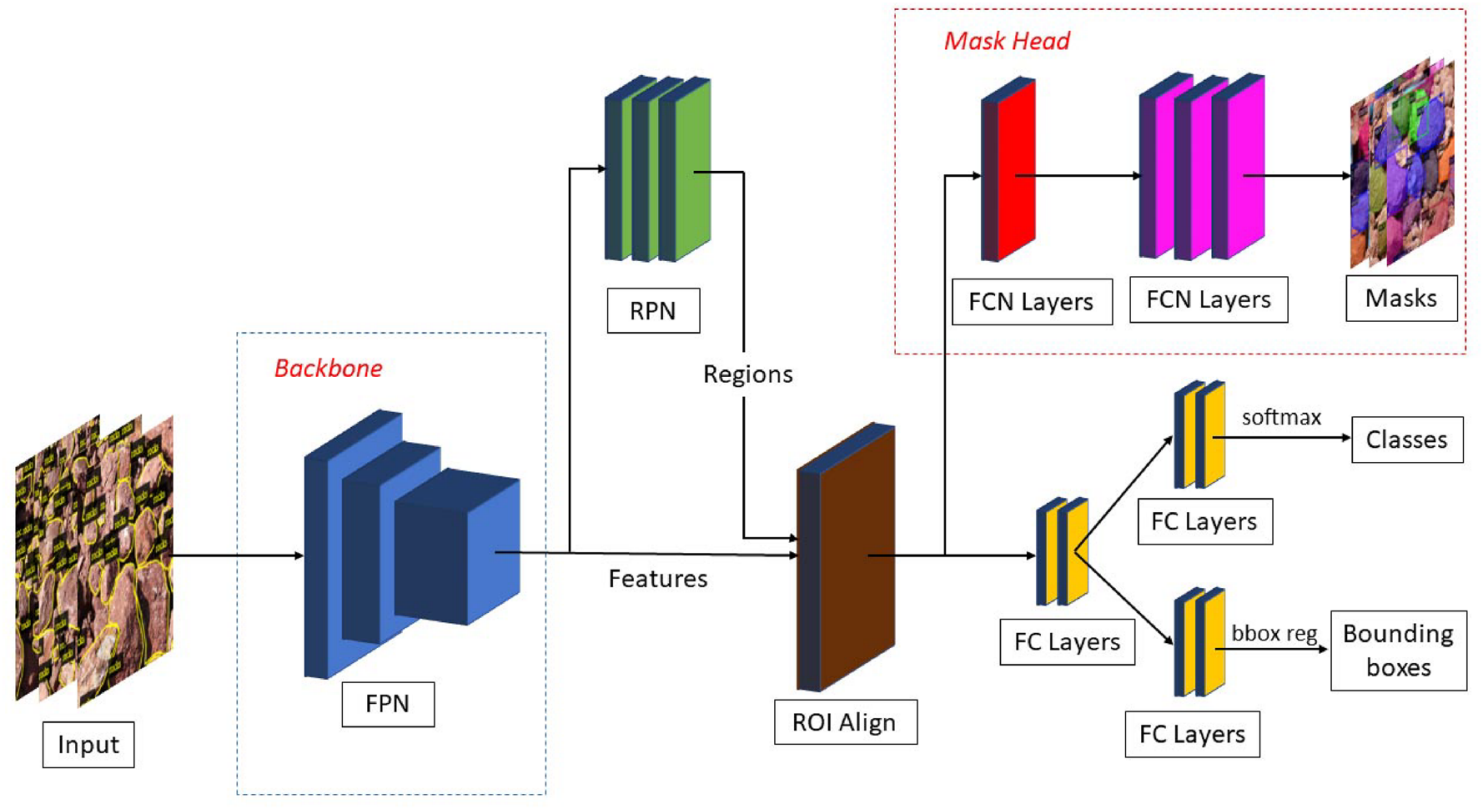
Framework of Mask R CNN with ResNet and FPN as backbone network

Mask R CNN is an instance segmentation which locates each pixel of every instances in an image. It is an extension of Faster R CNN^26^ which is used extensively for the object detection. Along with the localisation of the objects in the images an extra branch is added at the end of the model to parallelly predict the masks over the proposed bounding boxes. Mask R CNN uses Region Proposal Network (RPN) which was first introduced in Faster R CNN paper for proposing regions of interest and then classifying the proposals along with generating bounding boxes and masks. For the purpose of feature extraction, Feature Pyramid Network (FPN)^27^ is used with back bone network as ResNet50^28^ which extracts multi-scale feature maps. To warp the image features with the proposed ROIs, ROI Align is used instead of ROI Pooling which was used in Faster R CNN^23^. The output is fed to the Fully Connected (FC) and Fully Conventional Network (FCN)^29^ to generate the masks. The schematic diagram of CNN architecture is given in Fig. 3.

#### Feature extraction

Dump images contain many details of the particles like their texture, granularity, color, fines. The developed model needs to learn the features which will have the detailed information of each instance pixels. The choice of CNN for feature extraction is vital as the architecture, type and the depth of layers, number of parameters affects the accuracy, training time, detection speed and the overall performance of the Mask R CNN . The combination of ResNet and FPN is popularly used for extraction of features, as ResNet overcomes the degradation problem where accuracy gets saturated and often degrades with increase in depth of layers^28^. The FPN is a top down pyramid architecture which makes it possible to obtain multi scale features for better representing the target of different scales. FPN inputs dump images of 1024 $\times$ 1024 dimension and it consists of 5 stages for each different scale of feature maps. High level features extracted from the first pyramid are passed to second pyramid by lateral connections. Thus, each layer has access to both higher and lower level of features independently. In this study, Mask R CNN has been implemented using ResNet50 with FPN.

#### Generation of ROI

Extracted feature maps from different layers of backbone FPN is input to the RPN which was first introduced in Faster R CNN for the generation of regions of interests. RPN is a fully convolutional network which is responsible for proposing possible areas containing objects with bounding boxes and objectness score. 6 anchors of sizes: 16, 32, 64, 128, 256 and 512 with aspect ratios of 0.5, 1 and 2 has been defined for each size and distributed over entire dump image area. The output of RPN consists of category of anchors whether the anchor represents foreground (dump particle) or background. If the anchor contains the target, the coordinates of the selected anchor has to be adjusted to bound the target in a box. Therefore, the second output from the RPN contains the correction factor which defines the position and the size of proposed bounding boxes. Maximum of 2000 proposed bounding boxes with high objectness scores are selected. Non Max Suppression (NMS) with IOU threshold of 0.5 is applied to suppress highly overlapping RPN proposals based on their class score. Final proposals, which contain dump particles are sent to next stage.

#### ROI align and mask generation

The outputs from RPN and the backbone network are sent to Fully Connected (FC) and Fully Convolutional Network (FCN) for the target identification, localisation and mask generation. Before feeding the outputs to the next stage, the size of proposed anchor boxes needs to be adjusted as per the requirements of the FC network. The ROIs from RPN and the multi scale visual features from the backbone network are warped up by ROI Align operation which aligns the corresponding features of ROIs using bilinear interpolation^23^. Mask R CNN replaced ROI Pooling layer which was implemented in Faster R CNN with ROI Align layer which doesn’t uses quantization of features and it aligns each pixel of input to output and helps to produce better segmentation of pixels. The features from ROI Align layer is input to FC network for classification and box regression while the extra branch of FCN generates the masks of dump particles.

### Loss function and training of Mask R CNN

Loss function represents the differences in predicted values and ground truth values. The same multi task loss function as defined in original Mask R CNN paper^23^ has been implemented. It sums up the losses of classification, bounding box regression and segmentation masks as shown in Eq. (1).

1 $$\begin{aligned} L=L_{cls}+L_{bbox}+L_{mask} \end{aligned}$$

For implementing the Mask R CNN, the dump image dataset was split into three parts. The images with 80% of the total extracted particles were selected for training while the images with 10% each were selected for validation and test datasets. The ground truth data constituted 31,505 dump particles which were annotated under a single class named ”particle”. Before feeding the images in the model, dimensions of all images were fixed to 1024 $\times$ 1024 and the input batch size was 512 to train RPN so that the target foreground anchors would be more compared to a default value of 256. Learning rate of $2.5\times 10^{-4}$ was used with the decay of $10^{-3}$ in the normalization layers. Stochastic Gradient Descent (SGD) was used with a momentum of 0.9.

## Results

### Implementation details

The experiments were performed in Google Colab, a virtual computing platform that provides 25GB RAM of Tesla K80 GPU with storage of 68 GB. In this study, we have developed our own version of Mask R CNN based on Detectron2^30^, an open-source object detection system which is written in PyTorch^31^ framework for deep learning. The prepared dataset, consisting of the VIA generated ground truth segmentation masks of dump particles, was trained. The model weights were initialised with the weights obtained from a model that was trained on MSCOCO Dataset^25^, and training was done only for the network heads in all of our experiments by tuning its parameters to map the dump particles to ”particle” as a label. The model was trained to 10,000 iterations ,and it took 5.5 hours approximately. To prevent the overfitting of model, the dataset was augmented in several ways using random crops, random scaling, random translations, random rotations, adding Gaussian noise and random horizontal and vertical flips. The development of Mask R CNN based model is presented in two progressive steps. In the first experiment, 16,448 particles were used in the dataset with the backbone network of ResNet50 with FPN. In the second experiment, the number of particles in the dataset was increased to 31,505 particles. The performance of the model was analysed with an increasing number of particles in the dataset.

For evaluating the performance of our Mask R CNN based model, a mean percentage error for all the images of validation dataset is calculated between the areas of particles predicted from the model and from the annotated image that was given as an input to the model.

### Performance of Mask R CNN with ResNet50 as backbone network

In this study, performance of the model has been evaluated with the size of dataset.

#### On dataset containing 16,448 particles

**Figure 4:**
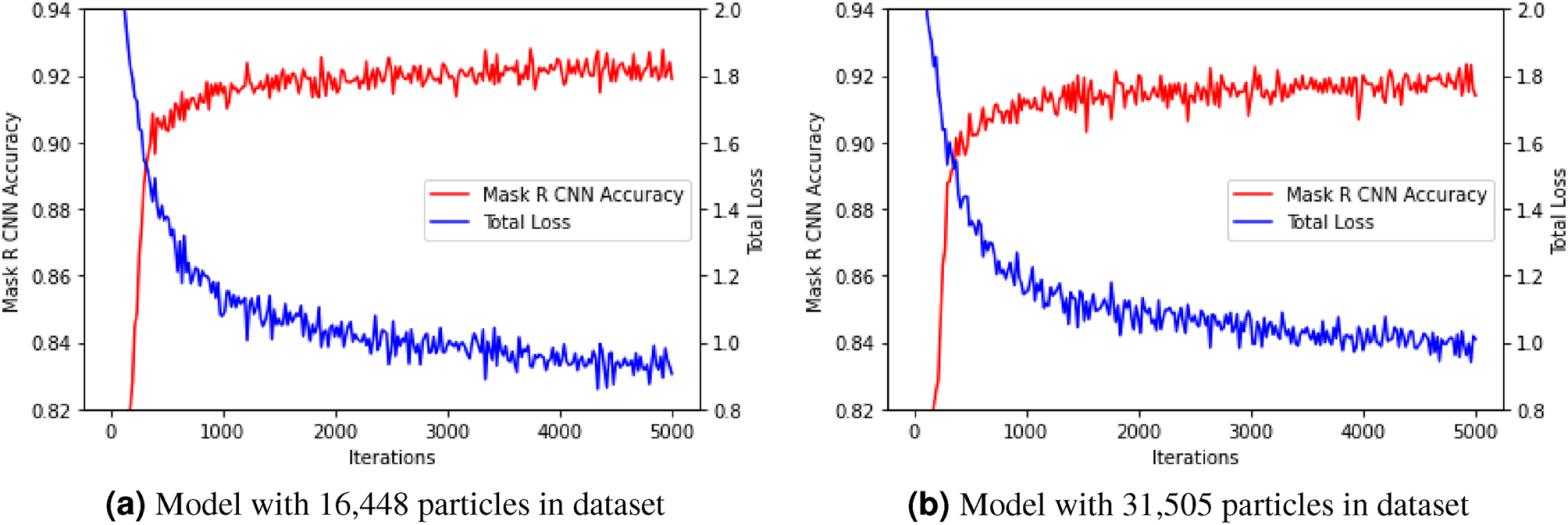
Plot of Mask R CNN training accuracy and Total Loss during training with steps of iterations for the model with ResNet50 plus FPN as backbone network

To accurately evaluate the quality of localization and segmentation of the dump particles, the Mask R CNN with backbone as ResNet50 plus an FPN was initially trained on the dump dataset containing 16,448 particles. Figure 4a shows the total loss and Mask R CNN training accuracy with the number of iterations in figure. Training started with a rapid downfall of total loss, and after 2000 steps of iteration the decrease of value for every 1000 iteration became 0.1. Training accuracy followed a reverse trend, and at the end of 7000 steps of iteration, the value was 94.3%. The training of the Mask R CNN model was evaluated by training accuracy and the total loss. The general strategy for calculating the training accuracy is to compare the predicted masks over the objects with the ground truth data at each iteration during training. In this case, the annotated masks of dump particles were compared with the predicted masks of the dump particles. If $\hat{\hbox {y}}_i$ is the predicted value of the $\hbox {i}^{\textrm{th}}$ sample and y$_i$ is the corresponding true value, then the fraction of correct predictions over $\hbox {n}_{\textrm{particles}}$ is defined as in Eq. (3)

2 $$\begin{aligned} z_i&= {\left\{ \begin{array}{ll} 1 \quad \text {if } ({\hat{y}}_i - y_i) = 0 \\ 0 \quad \text {if } ({\hat{y}}_i - y_i) \ne 0 \end{array}\right. } \end{aligned}$$

3 $$\begin{aligned} accuracy&= \frac{1}{n_{particles}}\sum _{i=0}^{n_{particles}-1}(z_i) \end{aligned}$$

The rate of false positives was decreasing very slowly with the number of iterations. The final value of Mask R CNN false positives at the end of 5000 steps of iteration was 0.1. The Mask R CNN model based on ResNet50 plus an FPN as a backbone network was able to identify multi scale dump particles. This experiment indicates that CNN, which has already obtained excellent results in other fields of computer vision, can also be helpful in the case of objects that are large in number, highly variable in shape, size, color, granularity and texture.

#### On dataset containing 31,505 particles

**Figure 5:**
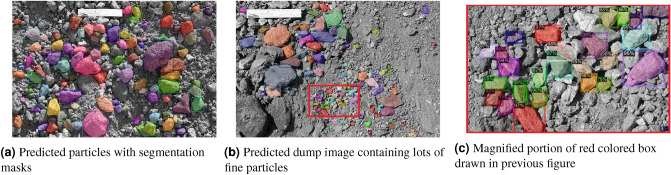
Inferences from the Mask R CNN with backbone network on ResNet50 plus an FPN

The next experiment was carried out on the dataset containing 31,505 particles with ResNet50 plus an FPN as the backbone network for the Mask R CNN model. All the other hyperparameters were kept as it was in the model with ResNet50 backbone network with dataset containing 16,448 dump particles. The output from the model had given a better segmentation of dump particles from the images. From the plot of training loss and Mask R CNN training accuracy as given in Fig. 4b, it was observed that there was an increase in the total loss with the same steps of iteration as compared to the model with dataset containing 16,448 particles. The training accuracy curve (calculated from Eq. 3) was observed to get flat in the initial phase of training. At the end of the training, the total loss was 0.86, and training accuracy was 97.2%. The trend of false positives was almost similar to the previous experimental result. The value of false positives was 0.11. The classification accuracy of this experiment was better than the previous one. The reason behind this behaviour is the increase in the size of training dataset which provided more number of images during the training of network. Some predicted dump images are shown in Fig. 5. Figure 5b shows a predicted dump image with lots of fine particles. Segmentation masks, bounding boxes, and prediction accuracy of individual particles are illustrated in Fig. 5c. The different colors of the masks show that these are different instances of a single object named “particle”.

### Calculation of percentage error between predicted and ground truth values

**Figure 6:**
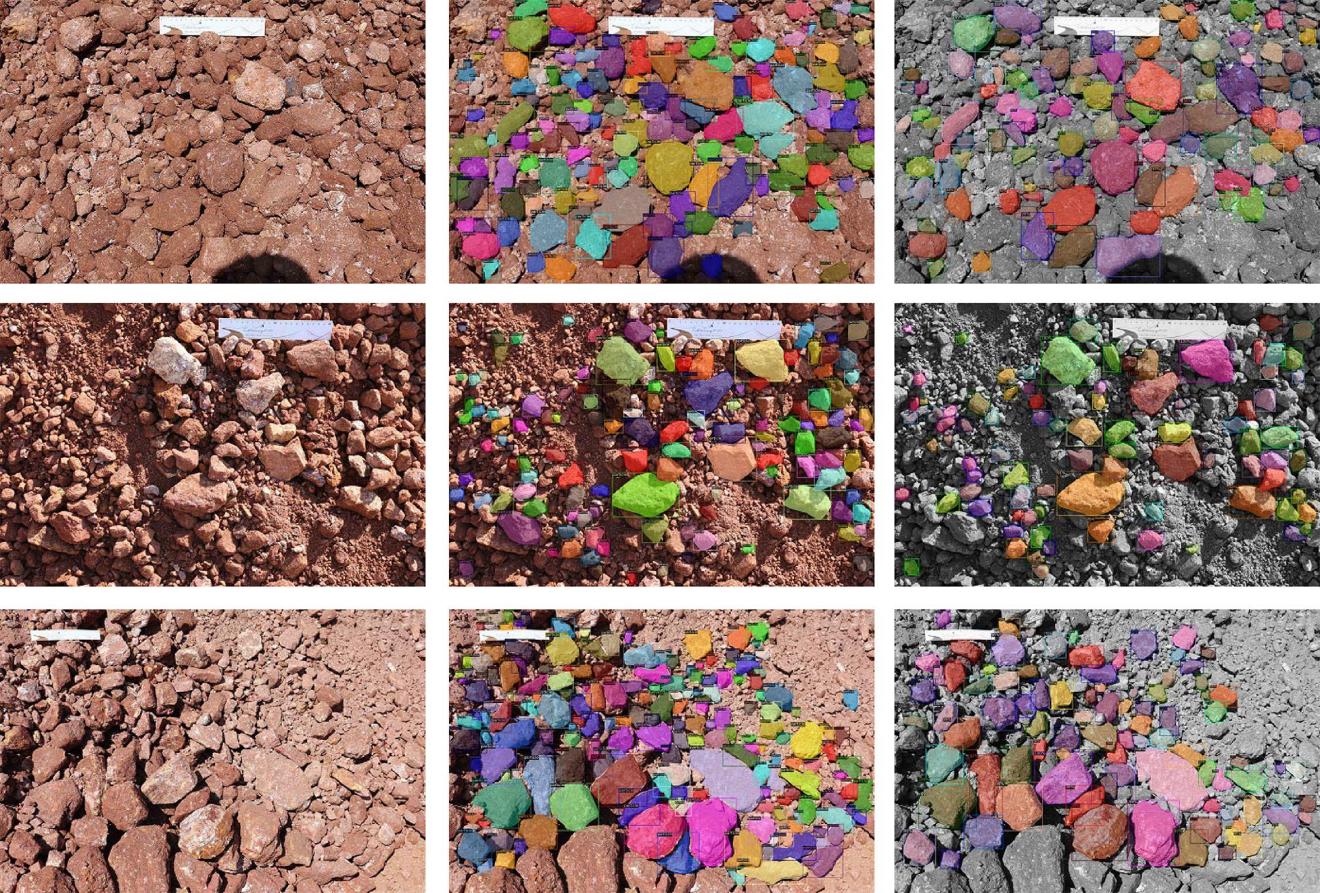
Original, ground truth and predicted results of image ids: IMG_9012, IMG_9078 and IMG_9301 in first, second and last rows respectively

From the output of the model with the backbone of ResNet50 plus an FPN on dump dataset containing 31,505 particles, the bounding box areas of dump particles were predicted, which will later be helpful in determining the particle size distribution curve and the other parameters that affect the stability of overburden dump. Figure 6 shows three sets of images containing original images from the validation dataset, labelled and annotated image fed to the model as input and predicted image respectively. For evaluating the predictions made by the model, we compared the bounding box areas of annotated particles with that of the predicted bounding box areas of the validation dataset. The annotations were recorded as polygons to calculate the bounding box from the polygon we first determined the coordinates of a minimum bounding rectangle over the polygon as per Eq. (4).

4 $$\begin{aligned} bbox = [min(x), max(x), min(y), max(y)] \end{aligned}$$

The area of the bounding box over a dump particle was then calculated as the area of the minimum bounding rectangle. The percentage error between the predicted and ground truth values was calculated as per Eq. (5).

5 $$\begin{aligned} Percent error (\%) = [\frac{1}{n_{particles}}\sum _{i=0}^{n_{particles}-1}\frac{(ar\_bbox_{gt})_i - (ar\_bbox_{pred})_i}{(ar\_bbox_{gt})_i}] \times 100 \end{aligned}$$

Here $n_{particles}$ is the number of particles common to the ground truth and predicted values of validation dataset. $ar\_bbox_{gt}$ and $ar\_bbox_{pred}$ are the area of bounding box of particle from ground truth and predicted mask respectively.

**Figure 7:**
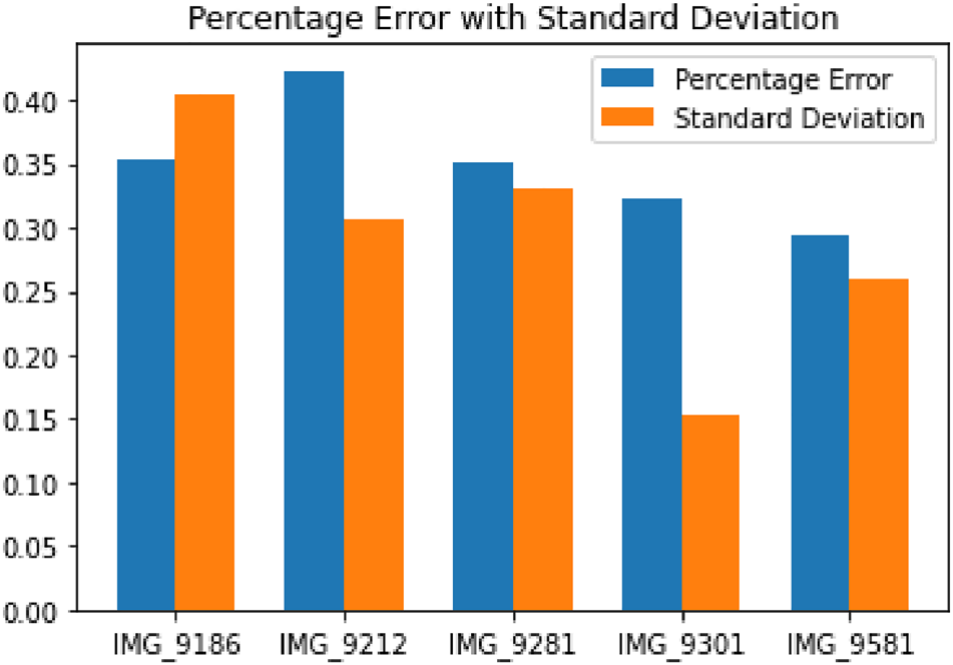
Percentage error with standard deviation between the bounding box areas from ground truth and predicted masks for 5 randomly selected images of validation dataset

It was observed that the areas of those particles which were segmented from the predicted images were in a close range with the areas of the same particles from ground truth data. A percentage error between the bounding box areas of particles that were common to both the ground truth data and the predicted values of the validation dataset was calculated. The bounding box areas of particles (in $(pixel)^2$) from ground truth data were calculated as per Eq. (4), and the bounding box area of particles (in $(pixel)^2$) were predicted from the model as shown in Fig. 5c. A mean percentage error was calculated as per Eq. (5) for all the common particles predicted in a dump image. Table 1 shows the bounding box areas of ground truth and predicted masks for 10 randomly selected particles of image: IMG_9281. Figure 7 shows the percentage error with standard deviation between the bounding box areas of dump particles from ground truth data and the predicted data for 5 randomly selected images from the validation dataset. The results obtained from the model are able to identify 73.58% particles on an average. A mean percentage error of 0.39% and standard deviation of 0.25 was found when the common particles from all the dump images of validation dataset were considered. Lower values of percentage error indicate that the classification and segmentation accuracy of the model was high, and it was able to delineate the dump particles accurately from the images. Therefore, the model is able to precisely locate the bounding boxes over dump particles.

**Table 1:** A table showing bounding box areas of 10 randomly selected dump particles of IMG_9281 from ground truth data and predicted masks of validation dataset

S. no.	$ar\_bbox_{gt}$ in $(pixel)^2$	$ar\_bbox_{pred}$ in $(pixel)^2$	Percent error (%)
1	330,099	330,427	0.1
2	3,173,703	3,175,804	0.07
3	1,695,358	1,702,061	0.4
4	983,766	982,579	0.12
5	16,548,080	16,557,954	0.06
6	4,357,080	4,364,645	0.17
7	10,980,008	11,131,568	1.38
8	1,908,522	1,897,543	0.58
9	769,775	769,080	0.09
10	2,409,304	2,424,045	0.61

## Discussion

This study shows that it is possible to use deep learning techniques even to detect dump particles which vary widely in shape, size, color, texture, and granularity. The images were collected from an active overburden dump, and the dump particles were annotated and labelled. Mask R CNN was implemented with ResNet50 plus an FPN as a backbone network. Two experiments were designed, the first one with 16,448 dump particles in the dataset, and in the second experiment, 15,057 more particles were added in the dataset. The model was trained to achieve an accuracy of 97.2% for the dataset containing 31,505 particles. Bounding box areas were predicted from the model with a mean percentage error of 0.39% with a standard deviation of 0.25. From the AI experimental results as shown in Fig. 6, it was found that the Mask R CNN model was able to predict similar size range of dump particles as annotated and labelled. We attempted to annotate as smaller particles as possible. The number of coarse particles was more than the fine particles in the prepared dump dataset. As a result, the trained model predicted a greater number of coarse particles. Therefore, it can be concluded that the trained Mask R CNN model is working better for coarse particles than fine particles. To address the fine particles problem, it is recommended to click the images by keeping camera closer to the dump slopes. As the white-colored scale is being used to transform the pixels into real-world coordinates, the fine particles would be captured and trained effectively. The images of a dumpsite can be captured at various positions perpendicular to the dump slope, and the predicted results can be merged to get the coarse and fine dump particles predicted by the model. The results will be better if high resolution dump images are used in the training dataset. However, it will be better to have more number of segmented particles to generate an accurate particle size distribution curve with less percentage of error.

## Conclusion

This study is the first step towards developing an AI-based tool to predict in situ particle size distribution of a section of a mine overburden dump using images. Particle size distribution plays an important role in controlling the shear strength of a dump. Once the behaviour of shear strength is established, the Factor of safety (FoS) can easily be anticipated. This work will help develop a tool that mine management can use to estimate the stability of dump instantaneously. The key point of relevance in this research work was to inquire about the size of dataset that would be sufficient for training the model to reach high training accuracy. If the dataset size is more than the images containing 31,505 particles, the training accuracy of the model may not improve significantly, as it has already reached 97.2% while if the size of dataset is smaller, then the desired results may not be obtained. A high training accuracy signifies the complexity involved in predicting objects varying widely in terms of shape, size, color, granularity, texture, etc. For undertaking this study, we prepared a small dataset of dump images containing 31,505 particles which have collections from a single mine. This kind of dataset is not available in the literature at present, so we will contribute the dump dataset to the research community in the future after adding more images from other mines of different ores. This dataset can also be used to study different concepts of overburden dumps like the gradation of particles, porosity of dump samples, distribution of particles based on their shape. Our primary focus will be on increasing the number of images in the dataset for future work. A large dataset will allow an increase in the depth of layers so that the model can be implemented with ResNet101 as the backbone network. Later, the particle size distribution curve can be determined for an unseen dump image from this model.

## Data Availability

The datasets used and/or analysed during the current study available from the corresponding author on reasonable request.

## References

[1] Wang G (2011). Research on particle size grading and slope stability analysis of super-high dumping site. Rock Soil Mech.

[2] Gao S (2017). Mechanical properties of material in a mine dump at the shengli# 1 surface coal mine, china. Int. J. Min. Sci. Technol..

[3] Chaulya S, Singh R, Chakraborty M, Dhar B (1999). Numerical modelling of biostabilisation for a coal mine overburden dump slope. Ecol. Model..

[4] Fernando, J. & Nag, D. A study of internal overburden dump design and stability analysis for hazelwood power mine, Latrobe Valley, Victoria, Australia. *Application of Computers and Operations Research in the Minerals Industries, South African Institute of Mining and Metallurgy* 267–274 (2003).

[5] Johari A, Khodaparast A (2015). Analytical stochastic analysis of seismic stability of infinite slope. Soil Dyn. Earthq. Eng..

[6] Iannacchione, A. T. & Vallejo, L. E. Shear strength evaluation of clay-rock mixtures. In *Slope Stability 2000*, 209–223 (ASCE LIBRARY, 2000).

[7] Xu W-J, Hu R-L, Tan R-J (2007). Some geomechanical properties of soil-rock mixtures in the hutiao gorge area, china. Geotechnique.

[8] Yellishetty M, Darlington WJ (2011). Effects of monsoonal rainfall on waste dump stability and respective geo-environmental issues: a case study. Environ. Earth Sci..

[9] Rahmani H, Scanlan C, Nadeem U, Bennamoun M, Bowles R (2019). Automated segmentation of gravel particles from depth images of gravel-soil mixtures. Comput. Geosci..

[10] Yaghoobi H, Mansouri H, Farsangi MAE, Nezamabadi-Pour H (2019). Determining the fragmented rock size distribution using textural feature extraction of images. Powder Technol..

[11] Thurley, M. J. Automated image segmentation and analysis of rock piles in an open-pit mine. In *2013 International Conference on Digital Image Computing: Techniques and Applications (DICTA)*, 1–8 (IEEE, 2013).

[12] Thurley MJ (2011). Automated online measurement of limestone particle size distributions using 3d range data. J. Process Control.

[13] Maerz NH, Palangio TC, Franklin JA (1996). Wipfrag image based granulometry system. Proc. FRAGBLAST.

[14] Liu, Q. & Tran, H. Comparing systems-validation of fragscan, wipfrag and split. *Measurement of Blast Fragmentation*, Franklin and Katsabanis (eds) 151–155 (1996).

[15] Anwar SM (2018). Medical image analysis using convolutional neural networks: a review. J. Med. Syst..

[16] Chen, X. *et al.* Monocular 3d object detection for autonomous driving. In *Proceedings of the IEEE Conference on Computer Vision and Pattern Recognition*, 2147–2156 (2016).

[17] Rawat W, Wang Z (2017). Deep convolutional neural networks for image classification: a comprehensive review. Neural Comput..

[18] Zhao Z-Q, Zheng P, Xu S-T, Wu X (2019). Object detection with deep learning: a review. IEEE Trans. Neural Netw. Learn. Syst..

[19] Garcia-Garcia, A., Orts-Escolano, S., Oprea, S., Villena-Martinez, V. & Garcia-Rodriguez, J. A review on deep learning techniques applied to semantic segmentation. arXiv preprint arXiv:1704.06857 (2017).

[20] Zeiler, M. D. & Fergus, R. Visualizing and understanding convolutional networks. In *European Conference on Computer Vision*, 818–833 (Springer, 2014).

[21] Karimpouli S, Tahmasebi P (2019). Segmentation of digital rock images using deep convolutional autoencoder networks. Comput. Geosci..

[22] Cheng, G. & Guo, W. Rock images classification by using deep convolution neural network. *J. Phys.: Conf. Ser.*, vol. 887 (IOP Publishing, 2017).

[23] He, K., Gkioxari, G., Dollár, P. & Girshick, R. Mask r-cnn. In *Proceedings of the IEEE International Conference on Computer Vision*, 2961–2969 (2017).

[24] Dutta, A., Gupta, A. & Zissermann, A. VGG image annotator (VIA). http://www.robots.ox.ac.uk/~vgg/software/via/ (2016).

[25] Lin, T.-Y. *et al.* Microsoft coco: Common objects in context. In *European Conference on Computer Vision*, 740–755 (Springer, 2014).

[26] Ren, S., He, K., Girshick, R. & Sun, J. Faster r-cnn: Towards real-time object detection with region proposal networks. In *Advances in Neural Information Processing Systems*, 91–99 (2015).

[27] Lin, T.-Y. *et al.* Feature pyramid networks for object detection. In *Proceedings of the IEEE Conference on Computer Vision and Pattern Recognition*, 2117–2125 (2017).

[28] He, K., Zhang, X., Ren, S. & Sun, J. Deep residual learning for image recognition. In *Proceedings of the IEEE Conference on Computer Vision and Pattern Recognition*, 770–778 (2016).

[29] Long, J., Shelhamer, E. & Darrell, T. Fully convolutional networks for semantic segmentation. In *Proceedings of the IEEE Conference on Computer Vision and Pattern Recognition*, 3431–3440 (2015). 10.1109/TPAMI.2016.2572683 27244717

[30] Wu, Y., Kirillov, A., Massa, F., Lo, W.-Y. & Girshick, R. Detectron2. https://github.com/facebookresearch/detectron2 (2019).

[31] Paszke, A. *et al.* Pytorch: An imperative style, high-performance deep learning library. In Wallach, H. *et al.* (eds.) *Advances in Neural Information Processing Systems, 32*, 8024–8035 (Curran Associates, Inc., 2019).

